# Evaluation of the relationship between inflammation and typical chest pain in ST-elevation myocardial infarction

**DOI:** 10.1186/s12014-026-09596-2

**Published:** 2026-03-11

**Authors:** Sophia Wolfermann, Timo Schmitz, Philip Raake, Jakob Linseisen, Christa Meisinger

**Affiliations:** 1 Chair of Epidemiology, Medical Faculty, University of Augsburg, Stenglinstraße 2, 86156 Augsburg, Germany; 2 Department of Cardiology, Respiratory Medicine and Intensive Care, University Hospital Augsburg, 86156 Augsburg, Germany

**Keywords:** Myocardial infarction, Chest pain, Inflammation, Protein markers, Registry

## Abstract

**Background:**

Previous investigations have shown that the absence of typical breast symptoms is associated with unfavorable outcomes after an acute myocardial infarction (AMI). Delayed diagnosis and therapy could not explain these results, so other causes seem to be involved. Therefore, in the present analysis the association between inflammatory plasma proteins and typical chest pain symptoms in hospitalized patients with acute ST-elevation myocardial infarction (STEMI) was investigated.

**Methods:**

Data from 395 STEMI patients registered by the population-based Myocardial Infarction Registry Augsburg between 2009 and 2013 were used for analysis. The OLINK inflammatory panel including a total of 92 cytokines was measured in arterial blood samples, which were obtained immediately after hospital admission within the scope of cardiac catheterization. The associations between the inflammation markers and typical chest pain were examined by multiple logistic regression analyses.

**Results:**

Altogether, 10.9% of the STEMI patients did not present with typical chest pain. The inflammatory markers IL8, IL6, FGF-21, CD40, CST5, ADA, OPG, PD-L1, TNFRSF9 and STAMBP were significantly inversely associated with typical chest pain after FDR-adjustment. The strongest associations were found for FGF-21, CST5 and CD40.

**Conclusions:**

These results suggest that a dysregulated inflammatory status is associated with a lack of typical chest pain in AMI patients. Beyond acute-phase inflammatory interleukins elevated within the early phase of an AMI, such as IL-6, hepatokines and transmembrane proteins seem to be associated with AMI symptoms. Further research into the causal mechanisms of these associations is necessary.

**Supplementary Information:**

The online version contains supplementary material available at 10.1186/s12014-026-09596-2.

## Background

The diagnosis of an acute myocardial infarction (AMI) is typically based on characteristic clinical symptoms and changes in the electrocardiogram (ECG). However, many patients experience atypical and non-specific symptoms when suffering an AMI [1]. This highlights the necessity of identifying additional parameters to ensure that this group of patients is also diagnosed quickly and accurately since prompt treatment of AMI is crucial for improving clinical outcomes and both short- and long-term survival [2, 3]. Given these diagnostic challenges, the study of biomarkers is gaining increasing importance in understanding the pathophysiological processes of AMI and developing new approaches for diagnostics and risk stratification. According to the World Health Organization (WHO), biomarkers are broadly defined as “any measurement reflecting an interaction between a biological system and an environmental agent, which may be chemical, physical, or biological” [4]. In the pathophysiology of AMI, ischemia caused primarily by thrombosis and plaque rupture plays a central role by activating inflammatory cells and triggering a signal transduction cascade that exacerbates tissue damage [5]. Some of the most well-known inflammatory biomarkers including C-reactive protein (CRP), interleukin-6 (IL-6), interleukin-1 (IL-1), and tumor necrosis factor-alpha (TNF-α) were shown to be associated with AMI and cardiac injury [5–7]. Since a persistent elevation of inflammatory biomarkers is associated with long-term myocardial damage after AMI, the identification of specific inflammatory markers in these patients could enable better prediction of long-term outcomes and early therapeutic intervention [8]. The advanced technologies available today enable rapid measurements of numerous pro-inflammatory and anti-inflammatory markers, which could provide information on the metabolic pathways involved and opportunities for the development of next-generation biomarkers. If laboratory parameters could serve as an additional diagnostic indicator particularly in AMI patients without typical symptoms, the detection of AMI could become more precise. In this study we investigated whether inflammatory and immune-related biomarkers are associated with typical chest pain in patients hospitalized with STEMI.

## Methods

### Study population

In the present study, data from the population-based Augsburg Myocardial Infarction Registry, Germany, were used. The registry was established in 1984 as part of the MONICA project (Monitoring Trends and Determinants in Cardiovascular Disease) and was continued between 1996 and 2000 as the KORA (Cooperative Health Research in the Augsburg Region) myocardial infarction registry. Since 2021, the registry has been based at Augsburg University Hospital. The study area covers around 700,000 inhabitants (city of Augsburg, county of Augsburg and Aichach-Friedberg). All patients aged 25 to 84 years with main residence in the study area who were admitted to one of the seven hospitals in the study area due to an AMI were continuously registered. Further information on case identification, diagnostic classification of events and quality control of data can be found in prior publications [9, 10]. The blood samples used for the present study were obtained from 398 consecutive patients who were admitted to Augsburg University Hospital between May 2009 and July 2013 due to a STEMI. All study participants gave written informed consent. The study (original data collection) was approved by the ethics committee of the Bavarian Medical Association (Bayerische Landesärztekammer), approval number 12057. Furthermore, the collection of blood was approved by the ethics committee of the Bavarian Medical Association, approval number 09016. The study and blood collection were performed in accordance with the Declaration of Helsinki.

### Data collection

During their hospital stay, all patients were interviewed by trained study nurses using a standardized questionnaire. Finally, the patients’ medical records were also reviewed to collect a variety of important medical data (including data on cardiovascular risk factors, medical history, concomitant diseases, medication, laboratory parameters and ECG). Typical chest pain was defined as chest pain or a feeling of pressure or tightness behind the breastbone.

### Blood collection and analysis

Of the 398 STEMI patients, blood samples were obtained during cardiac catheterization, which was usually performed immediately after hospital admission. The EDTA blood samples (arterial blood) were taken at the beginning of the catheterization and then immediately processed in the catheter laboratory (centrifugation, aliquoting and freezing at -80 °C). A panel of 92 inflammatory plasma proteins was measured in these patients.

For the measurement of the 92 proteins, the Proseek^®^ Multiplex Inflammation Panel (developed by Olink Proteomics, Uppsala, Sweden) was used. The measurements were based on the Proximity Extension Assay (PEA). More detailed information on the measurement can be found on the Olink Proteomics website [11] and in a previous publication [12]. A list of all measured plasma proteins including the short and long form names can be found in Table S1 of the supplementary material. According to OLINK specifications, all plasma proteins with 25% or more values below the limit of detection (LOD) were not considered for this analysis (17 proteins). For all other plasma proteins, we used the extrapolated values provided by OLINK, even if the value was below the LOD.

CRP levels were determined in venous blood samples taken at hospital admission (usually within 15 min) as part of the regular diagnosis and routine treatment. CRP-values were categorized into two groups (> 10 mg/dl versus ≤ 10 mg/dl). Glomerular filtration rate was estimated based on creatinine levels at admission by using the CKD-EPI formula [13].

### Statistical analysis

Categorical variables were presented as absolute frequencies with percentages and compared using Chi-square tests. Continuous variables were presented as mean and SD (standard deviation) or median and inter-quartile range (IQR) and compared using Student’s t tests or Mann–Whitney U tests. Boxplots were used to display group differences for plasma inflammation proteins and differences were tested using Student’s t tests.

### Logistic regression models

The obtained values for each plasma protein were standardized (the variable was centered and normalized so that the transformed variable had an expected value of 0 and variance of 1), which provides comparability between the 92 plasma proteins. Logistic regression models were calculated to examine the associations between the inflammatory plasma proteins (exposure) and the outcome typical chest pain (yes/no). According to literature research, the models were adjusted for sex (male/female), age (in years), renal function according to estimated GFR (3 groups: eGFR ≥ 60 ml/min/1.73m^2^, eGFR 30–59 ml/min/1.73m^2^, eGFR < 30 ml/min/1.73m^2^), diabetes mellitus (yes/no), and acute infection (CRP-values > 10 mg/dl versus < 10 mg/dl). False discovery rate (FDR)-adjustment of the obtained p-values was conducted to control for multiple testing. Outlier measurements that were 3 standard deviations or further from the mean were excluded from the analysis. The effect estimates (OR and 95% CI) of the logistic regression models must be interpreted as OR per standardized exposure associated with the outcome (binary variable).

### Sensitivity analysis

Based on 388 study participants, we examined whether the found associations were mainly independent of infarction size and therefore calculated the logistic regression models as explained above but additionally adjusted for troponin I (quantiles).

## Results

In three patients the information on plasma proteins was missing, so that a total of 395 AMI cases could be included in the present analysis.

The baseline characteristics for the total sample and stratified for typical chest pain (yes/no) in STEMI patients are given in Table 1. STEMI patients without typical chest pain were older, had more frequently a re-infarction, were less often current smokers, had more often low left ventricular ejection fraction (EF) values and impaired kidney function. Furthermore, they more often had CRP-values higher than 10 mg/dl at admission.

**Table 1:** Baseline characteristics for the total sample and by the presence of typical chest pain at acute STEMI (mean, SD; median IQR; n (%))

	Total sample	No Chest Pain	Chest Pain	*P*-Value	*N*
	*N* = 395	*N* = 43	*N* = 348		
Age (mean, SD)	63.5 (11.9)	68.6 (10.4)	63.0 (12.0)	0.002	395
Sex (female)	107 (27.1)	10 (23.3)	96 (27.6)	0.674	395
Prior myocardial infarction	42 (10.6)	9 (20.9)	33 (9.5)	0.043	395
**Comorbidities**					
Diabetes	107 (27.1)	16 (37.2)	89 (25.6)	0.149	395
Hypertension	301 (76.2)	34 (79.1)	263 (75.6)	0.751	395
Hyperlipidemia	218 (55.2)	17 (39.5)	198 (56.9)	0.046	395
**Smoking status**				< 0.001	395
Current smoker	156 (39.5)	11 (25.6)	143 (41.1)		
Ex smoker	106 (26.8)	9 (20.9)	97 (27.9)		
Never smoker	108 (27.3)	12 (27.9)	96 (27.6)		
Smoking status unknown	25 (6.3)	11 (25.6)	12 (3.4)		
**Left ventrucular ejection fraction**				0.001	395
EF > 50%	177 (44.8)	13 (30.2)	163 (46.8)		
EF 31–50%	173 (43.8)	18 (41.9)	155 (44.5)		
EF ≤ 30%	29 (7.3)	8 (18.6)	18 (5.2)		
EF unknown	16 (4.1)	4 ( 9.3)	12 (3.4)		
**Renal function**				< 0.001	395
eGFR ≥ 60 ml/min/1.73 m²	273 (69.1)	15 (34.9)	257 (73.9)		
eGFR 30–59 ml/min/1.73 m²	108 (27.3)	23 (53.5)	82 (23.6)		
eGFR < 30 ml/min/1.73 m²	14 (3.5)	5 (11.6)	9 (2.6)		
Admission CRP > 10 mg/dl	17 (4.3)	5 (11.9)	12 (3.5)	0.033	393
Troponin I at admission (ng/ml)	0.58 (0.1–5.9)	2.33 (0.3–7.9)	0.50 (0.1–4.9)	0.018	388
***Acute Treatment***					
Percutaneous coronary intervention (PCI)	360 (91.1)	37 (86.0)	320 (92.0)	0.312	395
Bypass surgery	41 (10.4)	4 (9.3)	37 (10.6)	0.996	395
Lysis	3 (0.8)	1 (2.3)	2 (0.6)	0.753	395

**Fig. 1:**
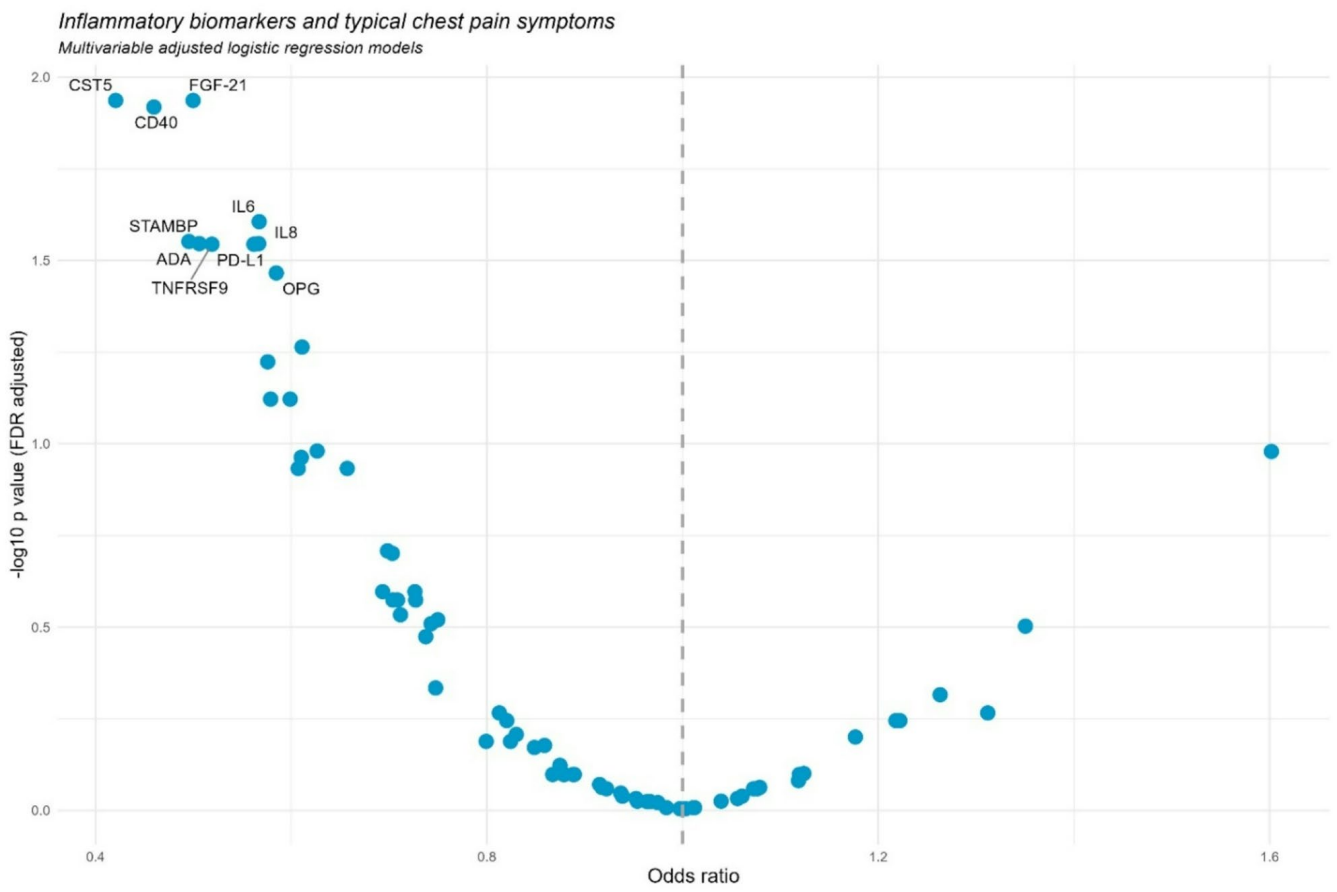
Results of the logistic regression models analyzing the association between typical chest pain and inflammatory plasma proteins. The models were adjusted for sex, age, renal function, diabetes, acute infection (CRP-values > 10 mg/dl). P-values were FDR-adjusted. Names of the markers are presented for all markers with FDR-adjusted p-values below 0.05

Results of the logistic regression analyses are displayed in Fig. 1. The plasma inflammation proteins IL8, IL6, FGF-21, CD40, CST5, ADA, PD-L1, STAMBP, TNFRSF9 and OPG were significantly inversely associated with typical chest pain after FDR-adjustment.

In Table S1 of the supplementary material, the total results (ORs, 95% CI, p values, FDR-adjusted p values) are displayed. This table also provides the full names of the measured plasma proteins. Table S2 (supplementary material) shows the median and IQR values for each plasma protein, stratified by typical chest pain symptoms. This table also lists all parameters excluded from regression analysis due to more than 25% values below the limit of detection. In particular, for FDF-23, there was a marked difference in median values between the two groups, with a highly significant result in the U-test.

**Fig. 2:**
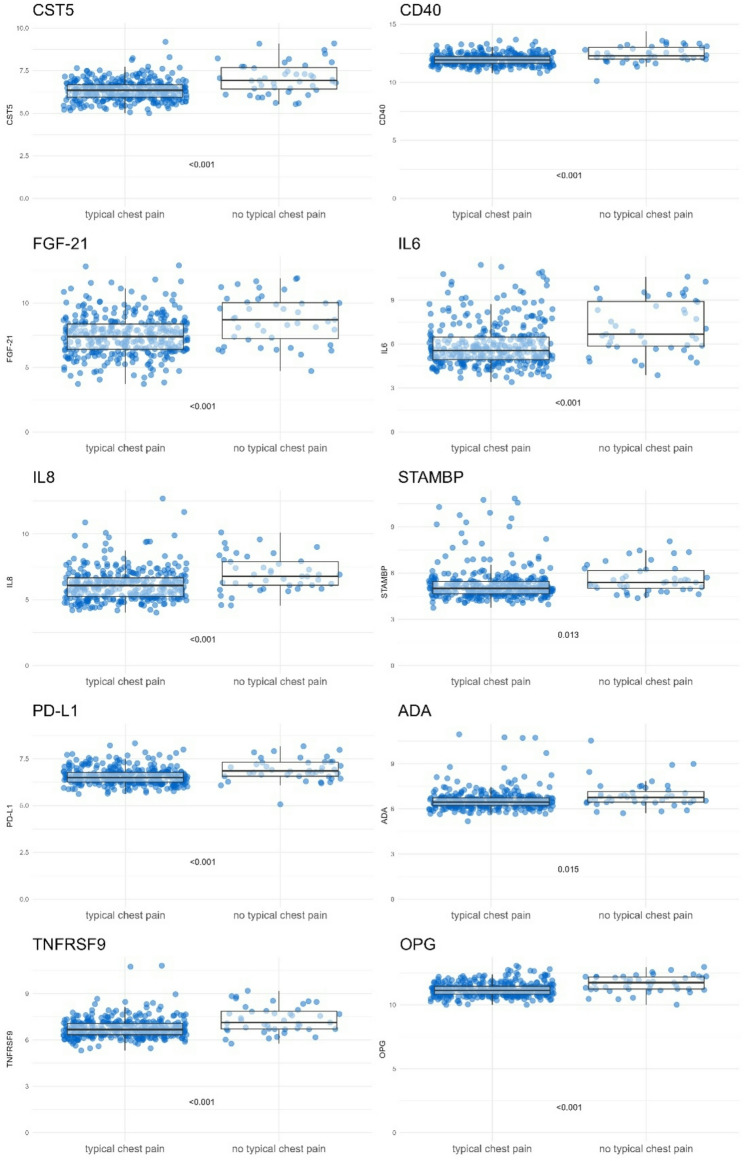
Boxplots of all inflammatory plasma proteins that were significantly associated in the logistic regression models, stratified for typical chest pain

Figure 2 shows the boxplots of plasma proteins that were significantly associated with chest pain in the regression analysis. The plots were stratified by typical chest pain symptoms. For each protein that was associated in the regression analysis, the unadjusted values were significantly higher in the ‘no chest pain’ group compared to the ‘typical chest pain’ group.

Figure S1 displays the results of the sensitivity analysis, in which regression models were additionally adjusted for troponin quantiles. This had almost no influence on the found effect sizes, although the p values were larger than in the original models.

## Discussion

In this analysis, the inflammatory markers IL8, IL6, FGF-21, CD40, CST5, ADA, OPG, PD-L1, TNFRSF9 and STAMBP at admission were significantly inversely associated in AMI patients with typical chest pain after FDR-adjustment. The strongest associations were found for FGF-21, CST5 and CD40.

The diagnosis of an AMI traditionally relies on the presence of typical symptoms, electrocardiographic changes, and the detection of cardiac biomarkers [14]. Myocardial cell death typically begins within six hours after the onset of ischemia [15, 16]. Since all forms of myocardial injury trigger an immunological cascade, this opens up both diagnostic and therapeutic perspectives [17], so that the diagnostic importance of cardiac biomarkers has significantly increased in recent years [14]. Various inflammatory markers can be detected during AMI, which may be classified according to their origin into three categories: biomarkers that were elevated prior to infarction; those directly released from the damaged myocardium; and systemic markers that rise in response to the ischemic event [4, 18]. In particular, markers from the third group, may provide insights into the patient’s symptomatic experience during MI and play a role in diagnosis, therapy, and prognosis [18]. Among the acute-phase inflammatory markers, CRP is of note; it correlates with IL-6 and typically peaks within the first two days after an AMI [8, 15]. IL-6 is a pleiotropic cytokine with broad biological functions, affecting both the innate and adaptive immune systems, as well as exerting direct effects on target tissues including the myocardium [19, 20]. Furthermore, IL-6 exhibits a dual role: it is cardioprotective in the acute phase, while chronically elevated levels are associated with adverse structural and functional myocardial changes [19, 21]. Elevated IL-6 levels have also been associated with increased cardiovascular risk prior to infarction, with some studies indicating more than a twofold increase in the risk of coronary artery disease [19, 21, 22]. In the present analysis, elevated IL-6 levels were observed among patients reporting less pronounced symptoms. The underlying mechanisms remain incompletely understood. Acutely, IL-6 is secreted by cardiomyocytes in response to ischemia and exerts anti-apoptotic and tissue-repairing effects [19]. At the same time, IL-6 has been shown to reduce myocardial contractility and sympathetic activity, potentially leading to an attenuated physiological response, manifesting as fewer or less intense symptoms such as chest pain or dyspnea [19]. Alternatively, elevated IL-6 levels may have been preexisting, reflecting chronic comorbid conditions such as atherosclerosis or type 2 diabetes mellitus—both of which are associated with a higher likelihood of “silent” myocardial infarction [15, 23]. The long-term prognostic significance of IL-6 is also well established, with persistently elevated levels associated with poorer outcomes following MI, a finding reflected in a previous study of our group [19].

Another key inflammatory pathway identified in this study involves the cluster of differentiation 40 (CD40) protein, which interacts with its ligand (CD40L). CD40L is primarily expressed on activated platelets and plays a central role in both inflammatory and prothrombotic processes [15, 24]. In conjunction with TNF-α, which itself promotes the production of IL-6 and matrix metalloproteinases, this pathway contributes to plaque destabilization and ischemic progression [15].

High levels of the pro-inflammatory cytokine IL-8, which was also inversely related to typical chest pain in our investigation, were associated with larger infarct size, impaired left ventricular function recovery, and adverse clinical outcomes in a prior study on STEMI patients [25]. In addition, IL-8 seems to be associated with left ventricular remodeling after acute myocardial infarction [26].

We also found a strong inverse association between typical chest pain and the levels of Fibroblast Growth Factor 21 (FGF-21), another marker known to increase early after an AMI. Primarily secreted by the liver under physiological conditions, FGF-21 exhibits antioxidant and anti-apoptotic properties [27]. It typically peaks around 24 h post-infarction and remains elevated for up to seven days [27]. Its cardioprotective effects are partly due to suppression of IL-6, TNF-α, and reactive oxygen species (ROS), which may explain the observation that patients with higher FGF-21 levels experienced milder symptoms despite ongoing myocardial injury [27, 28]. At the same time, FGF-21 positively correlates with markers of organ stress (e.g. AST, creatinine, NT-proBNP), underscoring its relevance as a prognostic indicator [29].

Other biomarkers significantly inversely associated with typical chest pain in this study included Cystatin D (CST5), adenosine deaminase (ADA), Programmed cell death 1 ligand 1 (PD-L1), STAM-binding protein (STAMPB), Osteoprotegerin (OPG) and TNF receptor superfamily member 9 (TNFRSF9), also known as CD137. The enzyme ADA belongs to the purine metabolism, and its activity in serum is considered a marker for AMI-related inflammation [30]. Changes in ADA activity have been demonstrated in a number of cardiovascular diseases such as atherosclerosis, myocardial infarction, hypertension, diabetes or thrombosis [31].

Several studies have shown that CD137 signalling is involved in the regulation of a variety of cell death methods, including apoptosis and autophagy [32, 33], and it has been observed that CD137 is expressed in human atherosclerotic plaque and patients with acute coronary syndrome [34, 35]. Elevated levels of soluble CD137 (sCD137) have been identified as an independent risk factor for ischemia-reperfusion injury in patients with STEMI [36]. Furthermore, CD137 deficiency has been shown to protect against post-infarction cardiac fibrosis and adverse remodeling by modulating the ERK1/2 signaling pathway [37].

CST5, a cysteine protease inhibitor, has a role in regulating gene transcription and protein expression beyond its protease inhibitory function [38]. No prior study investigated whether there is an association between CST5 and AMI symptoms, however in a study by Schmitz et al. a relationship of CST5 with increased 28-day mortality following acute MI was found [39].

PD-L1 is a surface protein that plays an important role in inhibition of immune responses and is known to be involved in cancer development and therapy [40]. Prior literature indicates that it might be involved in ischemic and non-ischemic heart failure [41]. The PD-1–PD-L1 pathway could also be important in the regulation of cardiac regeneration [42]. Miyazaki et al. reported that in coronary artery disease patients, high soluble PD-L1 levels were associated with future cardiovascular events [43].

OPG is a soluble receptor and part of the TNF receptor family. It is primarily involved in the regulation of bone metabolism [44]. In a review by Samadi et al. it was suggested that OPG might be associated with an increased risk of coronary artery calcification [45]. Furthermore, OPG levels are associated with the severity of coronary artery disease (CAD) [46] and (long-term) prognosis after coronary artery disease [47, 48].

STAMPB is a relatively unexplored protein which is part of signal transduction in the JAK-STAT cascade [49, 50]. To our knowledge, there is only very limited research of its involvement in cardiovascular diseases in general and myocardial infarction in particular. In a small study by Björkenheim et al. (*n* = 24) it was found that the sweat of STEMI patients contained significantly higher levels of STAMPB compared to controls [51].

A recent study showed that patients presenting without most typical MI symptoms face a higher risk of short-term mortality [31]. In terms of long-term prognosis, most AMI symptoms were linked to a decreased mortality risk; however, shortness of breath and syncope/unconsciousness stood out by being associated with higher long-term mortality [52]. It is now well established that the cardiovascular and immune systems are intimately linked [53]. While inflammatory markers may exert symptom-dampening effects in the acute phase, some are simultaneously associated with worse short or long-term prognosis [19, 21, 29, 39, 43, 54, 55]. Despite promising findings, many of these associations remain incompletely understood, highlighting the need for further research into their causal mechanisms.

In this study, 17 proteins had to be excluded from the analyses due to 25% or more values below the LOD. Among them, IL-20, IL-33, leukemia inhibitory factor (LIF), and fibroblast growth factor 23 (FGF-23) differed significantly between patients with and without typical chest pain. The largest difference was seen for FGF-23, with higher median (IQR) levels in patients without typical chest pain (2.34 [1.32–2.65]) than in those with typical chest pain (1.30 [0.98–1.53]; *p* < 0.001). Prior studies showed that FGF-23 is frequently elevated in AMI, likely due to systemic inflammation, neurohormonal activation (particularly of the renin–angiotensin–aldosterone system), myocardial stress, and renal dysfunction [56, 57]. Elevated FGF-23 concentrations are independently associated with adverse left ventricular remodeling, reduced ejection fraction, heart failure, increased long-term mortality [58] and recurrent cardiovascular events [59–61]. Further research is needed to determine whether excluded proteins, in particular FGF-23, along with the identified inflammatory markers are associated with typical chest pain and may improve diagnosis of AMI.

### Strengths and limitations

This study has several strengths. The analyses were based on data from the population-based Augsburg Myocardial Infarction Registry with ongoing recruitment of patients, which minimizes the effects of selection bias. Blood samples were collected uniformly during the cardiac catheterization procedure, ensuring a standardized approach to blood collection. Extensive information was available for each AMI case, which could be used for appropriate adjustment in the logistic regression models. However, there are also some limitations. This study is based on observational data only, so no conclusions on causality can be drawn (including the possibility of reverse causality). In addition, residual confounding and unmeasured confounding cannot be completely ruled out. The associations found in this study could not be validated in another cohort of AMI patients. Only men and women between 25 and 84 years of age with STEMI were included in the analysis, so the results cannot be generalized to all age or ethnic groups or to non-ST-elevation MIs.

## Conclusions

The absence of typical chest pain symptoms associated with an AMI appears to trigger a systemic inflammatory response. This inflammatory process includes the release of cytokines such as IL-6 and IL-8 as well as hepatokines and transmembrane proteins. The strongest associations were found with the transmembrane proteins FGF-21, CST5 and CD40. Whether these inflammatory markers and the underlying pathways involved prove to be suitable for improving the diagnosis of AMI in patients without typical symptoms must be investigated in further studies.

## Supplementary Information

Supplementary Material 1

## Data Availability

The data underlying this article cannot be shared publicly because the data are subject to national data protection laws and restrictions that were imposed by the ethics committee of the Bavarian Medical Association (“Bayerische Landesärztekammer”) to ensure data privacy of the study participants because they did not explicitly consent to the data being made publicly available. The data will be shared at reasonable requests to the corresponding author.

## References

[1] DeVon HA, Mirzaei S, Zègre-Hemsey J. Typical and Atypical Symptoms of Acute Coronary Syndrome: Time to Retire the Terms? J Am Heart Assoc. 2020;9:e015539. 10.1161/JAHA.119.015539. 32208828 10.1161/JAHA.119.015539 PMC7428604

[2] Huang X, Bai S, Luo Y. Advances in research on biomarkers associated with acute myocardial infarction: A review. Med (Baltim). 2024;103:e37793. 10.1097/MD.0000000000037793. 10.1097/MD.0000000000037793 PMC11018244 38608048

[3] Odeberg J, Halling A, Ringborn M, Freitag M, Persson ML, Vaara I, et al. Markers of inflammation predicts long-term mortality in patients with acute coronary syndrome - a cohort study. BMC Cardiovasc Disord. 2025;25:190. 10.1186/s12872-025-04608-9. 40089663 10.1186/s12872-025-04608-9 PMC11909928

[4] International Programme on Chemical Safety. Biomarkers in risk assessment: Concepts and principles. Geneva: World Health Organization; 1993.

[5] Saxena A, Russo I, Frangogiannis NG. Inflammation as a therapeutic target in myocardial infarction: learning from past failures to meet future challenges. Translational research: J Lab Clin Med. 2016;167:152–66. 10.1016/j.trsl.2015.07.002. 10.1016/j.trsl.2015.07.002 PMC4684426 26241027

[6] Anderson DR, Poterucha JT, Mikuls TR, Duryee MJ, Garvin RP, Klassen LW, et al. IL-6 and its receptors in coronary artery disease and acute myocardial infarction. Cytokine. 2013;62:395–400. 10.1016/j.cyto.2013.03.020. 23582716 10.1016/j.cyto.2013.03.020

[7] Neri M, Fineschi V, Di Paolo M, Pomara C, Riezzo I, Turillazzi E, Cerretani D. Cardiac oxidative stress and inflammatory cytokines response after myocardial infarction. Curr Vasc Pharmacol. 2015;13:26–36. 10.2174/15701611113119990003. 23628007 10.2174/15701611113119990003

[8] Gabriel AS, Martinsson A, Wretlind B, Ahnve S. IL-6 levels in acute and post myocardial infarction: their relation to CRP levels, infarction size, left ventricular systolic function, and heart failure. Eur J Intern Med. 2004;15:523–8. 10.1016/j.ejim.2004.07.013. 15668089 10.1016/j.ejim.2004.07.013

[9] Löwel H, Meisinger C, Heier M, Hörmann A. The population-based acute myocardial infarction (AMI) registry of the MONICA/KORA study region of Augsburg. Gesundheitswesen. 2005;67(Suppl 1):S31–7. 10.1055/s-2005-858241. 16032515 10.1055/s-2005-858241

[10] Kuch B, Heier M, von Scheidt W, Kling B, Hoermann A, Meisinger C. 20-year trends in clinical characteristics, therapy and short-term prognosis in acute myocardial infarction according to presenting electrocardiogram: the MONICA/KORA AMI Registry (1985–2004). J Intern Med. 2008;264:254–64. 10.1111/j.1365-2796.2008.01956.x. 18397247 10.1111/j.1365-2796.2008.01956.x

[11] Olink Proteomics. Olink Proteomics. Normalized protein eXpression. https://olink.com

[12] Ponce-de-Leon M, Linseisen J, Peters A, Linkohr B, Heier M, Grallert H, et al. Novel associations between inflammation-related proteins and adiposity: A targeted proteomics approach across four population-based studies. Translational research: J Lab Clin Med. 2022;242:93–104. 10.1016/j.trsl.2021.11.004. 10.1016/j.trsl.2021.11.004 34780968

[13] Levey AS, Stevens LA, Schmid CH, Zhang YL, Castro AF, Feldman HI, et al. A new equation to estimate glomerular filtration rate. Ann Intern Med. 2009;150:604–12. 10.7326/0003-4819-150-9-200905050-00006. 19414839 10.7326/0003-4819-150-9-200905050-00006 PMC2763564

[14] Alpert JS, Thygesen K, Antman E, Bassand JP. Myocardial infarction redefined–a consensus document of The Joint European Society of Cardiology/American College of Cardiology Committee for the redefinition of myocardial infarction. J Am Coll Cardiol. 2000;36:959–69. 10.1016/s0735-1097(00)00804-4. 10987628 10.1016/s0735-1097(00)00804-4

[15] Khalil H. Traditional and novel diagnostic biomarkers for acute myocardial infarction. Egypt J Intern Med. 2022. 10.1186/s43162-022-00178-w.

[16] Mythili S, Malathi N. Diagnostic markers of acute myocardial infarction. Biomed Rep. 2015;3:743–8. 10.3892/br.2015.500. 26623010 10.3892/br.2015.500 PMC4660641

[17] Hofmann U, Frantz S. Neue Aspekte für kardiale Diagnostik und Therapie: Inflammation bei Herzinsuffizienz und Infarkt. Deutsches Ärzteblatt Online. 2022. 10.3238/PersKardio.2022.09.16.02.

[18] Wu Y, Pan N, An Y, Xu M, Tan L, Zhang L. Diagnostic and Prognostic Biomarkers for Myocardial Infarction. Front Cardiovasc Med. 2020;7:617277. 10.3389/fcvm.2020.617277. 33614740 10.3389/fcvm.2020.617277 PMC7886815

[19] Fontes JA, Rose NR, Čiháková D. The varying faces of IL-6: From cardiac protection to cardiac failure. Cytokine. 2015;74:62–8. 10.1016/j.cyto.2014.12.024. 25649043 10.1016/j.cyto.2014.12.024 PMC4677779

[20] Tanaka T, Narazaki M, Kishimoto T. IL-6 in inflammation, immunity, and disease. Cold Spring Harb Perspect Biol. 2014;6:a016295. 10.1101/cshperspect.a016295. 25190079 10.1101/cshperspect.a016295 PMC4176007

[21] Ridker PM. Inhibiting Interleukin-6 to Reduce Cardiovascular Event Rates: A Next Step for Atherothrombosis Treatment and Prevention. J Am Coll Cardiol. 2021;77:1856–8. 10.1016/j.jacc.2021.02.060. 33858621 10.1016/j.jacc.2021.02.060

[22] Danesh J, Kaptoge S, Mann AG, Sarwar N, Wood A, Angleman SB, et al. Long-term interleukin-6 levels and subsequent risk of coronary heart disease: two new prospective studies and a systematic review. PLoS Med. 2008;5:e78. 10.1371/journal.pmed.0050078. 18399716 10.1371/journal.pmed.0050078 PMC2288623

[23] Chiariello M, Indolfi C. Silent myocardial ischemia in patients with diabetes mellitus. Circulation. 1996;93:2089–91. 10.1161/01.cir.93.12.2089. 8925575 10.1161/01.cir.93.12.2089

[24] Daub S, Lutgens E, Münzel T, Daiber A. CD40/CD40L and Related Signaling Pathways in Cardiovascular Health and Disease-The Pros and Cons for Cardioprotection. Int J Mol Sci. 2020. 10.3390/ijms21228533. 33198327 10.3390/ijms21228533 PMC7697597

[25] Shetelig C, Limalanathan S, Hoffmann P, Seljeflot I, Gran JM, Eritsland J, Andersen GØ. Association of IL-8 With Infarct Size and Clinical Outcomes in Patients With STEMI. J Am Coll Cardiol. 2018;72:187–98. 10.1016/j.jacc.2018.04.053. 29976293 10.1016/j.jacc.2018.04.053

[26] Zarrouk-Mahjoub S, Zaghdoudi M, Amira Z, Chebi H, Khabouchi N, Finsterer J, et al. Pro- and anti-inflammatory cytokines in post-infarction left ventricular remodeling. Int J Cardiol. 2016;221:632–6. 10.1016/j.ijcard.2016.07.073. 27423081 10.1016/j.ijcard.2016.07.073

[27] Zhang Y, Liu D, Long X-X, Fang Q-C, Jia W-P, Li H-T. The role of FGF21 in the pathogenesis of cardiovascular disease. Chin Med J (Engl). 2021;134:2931–43. 10.1097/CM9.0000000000001890. 34939977 10.1097/CM9.0000000000001890 PMC8710326

[28] Bugger H, Pfeil K. Mitochondrial ROS in myocardial ischemia reperfusion and remodeling. Biochim Biophys Acta Mol Basis Dis. 2020;1866:165768. 10.1016/j.bbadis.2020.165768. 32173461 10.1016/j.bbadis.2020.165768

[29] Wu G, Wu S, Yan J, Gao S, Zhu J, Yue M, et al. Fibroblast Growth Factor 21 Predicts Short-Term Prognosis in Patients With Acute Heart Failure: A Prospective Cohort Study. Front Cardiovasc Med. 2022;9:834967. 10.3389/fcvm.2022.834967. 35369322 10.3389/fcvm.2022.834967 PMC8965840

[30] Patil N, Chavan V, Karnik ND. Antioxidant status in patients with acute myocardial infarction. Indian J Clin Biochem. 2007;22:45–51. 10.1007/BF02912880. 23105651 10.1007/BF02912880 PMC3454265

[31] Kutryb-Zajac B, Mierzejewska P, Slominska EM, Smolenski RT. Therapeutic Perspectives of Adenosine Deaminase Inhibition in Cardiovascular Diseases. Molecules. 2020. 10.3390/molecules25204652. 33053898 10.3390/molecules25204652 PMC7587364

[32] Xu Y, Zhang Y, Xu Y, Zang G, Li B, Xia H, Yuan W. Activation of CD137 signaling promotes macrophage apoptosis dependent on p38 MAPK pathway-mediated mitochondrial fission. Int J Biochem Cell Biol. 2021;136:106003. 10.1016/j.biocel.2021.106003. 33971320 10.1016/j.biocel.2021.106003

[33] Chen R, Xu Y, Zhong W, Li B, Yang P, Wang ZQ, et al. Activation of CD137 Signaling Enhances Vascular Calcification through c-Jun N-Terminal Kinase-Dependent Disruption of Autophagic Flux. Mediators Inflamm. 2018;2018:8407137. 10.1155/2018/8407137. 30356425 10.1155/2018/8407137 PMC6178178

[34] Olofsson PS, Söderström LA, Wågsäter D, Sheikine Y, Ocaya P, Lang F, et al. CD137 is expressed in human atherosclerosis and promotes development of plaque inflammation in hypercholesterolemic mice. Circulation. 2008;117:1292–301. 10.1161/CIRCULATIONAHA.107.699173. 18285570 10.1161/CIRCULATIONAHA.107.699173

[35] Dongming L, Zuxun L, Liangjie X, Biao W, Ping Y. Enhanced levels of soluble and membrane-bound CD137 levels in patients with acute coronary syndromes. Clin Chim Acta. 2010;411:406–10. 10.1016/j.cca.2009.12.011. 20026323 10.1016/j.cca.2009.12.011

[36] Pan YJ, Chen R, Xu Y, Xia H, Xu C, Yuan W. Association between CD137 and ischemia-reperfusion injury in patients with acute ST-segment elevation myocardial infarction. Zhonghua xin xue guan bing za zhi. 2021;49:1198–205. 10.3760/cma.j.cn112148-20210517-00425. 34905897 10.3760/cma.j.cn112148-20210517-00425

[37] Zang G, Chen Y, Guo G, Wan A, Li B, Wang Z. Protective Effect of CD137 Deficiency Against Postinfarction Cardiac Fibrosis and Adverse Cardiac Remodeling by ERK1/2 Signaling Pathways. J Cardiovasc Pharmacol. 2024;83:446–56. 10.1097/FJC.0000000000001549. 38416872 10.1097/FJC.0000000000001549

[38] Ferrer-Mayorga G, Alvarez-Díaz S, Valle N, de Las Rivas J, Mendes M, Barderas R, et al. Cystatin D Locates in the Nucleus at Sites of Active Transcription and Modulates Gene and Protein Expression *. J Biol Chem. 2015;290:26533–48. 10.1074/jbc.M115.660175. 26364852 10.1074/jbc.M115.660175 PMC4646312

[39] Schmitz T, Harmel E, Heier M, Peters A, Linseisen J, Meisinger C. Inflammatory plasma proteins predict short-term mortality in patients with an acute myocardial infarction. J Transl Med. 2022;20:457. 10.1186/s12967-022-03644-9. 36209229 10.1186/s12967-022-03644-9 PMC9547640

[40] Gou Q, Dong C, Xu H, Khan B, Jin J, Liu Q, et al. PD-L1 degradation pathway and immunotherapy for cancer. Cell Death Dis. 2020;11:955. 10.1038/s41419-020-03140-2. 33159034 10.1038/s41419-020-03140-2 PMC7648632

[41] Kushnareva E, Kushnarev V, Artemyeva A, Mitrofanova L, Moiseeva O. Myocardial PD-L1 Expression in Patients With Ischemic and Non-ischemic Heart Failure. Front Cardiovasc Med. 2021;8:759972. 10.3389/fcvm.2021.759972. 35096992 10.3389/fcvm.2021.759972 PMC8792535

[42] Qin J, Huang GN, Moslehi J. PD-1-PD-L1 immunomodulatory pathway regulates cardiac regeneration. Nat Cardiovasc Res. 2024;3:410–1. 10.1038/s44161-024-00461-9. 39196218 10.1038/s44161-024-00461-9

[43] Miyazaki S, Fujisue K, Yamanaga K, Sueta D, Usuku H, Tabata N, et al. Prognostic Significance of Soluble PD-L1 on Cardiovascular Outcomes in Patients with Coronary Artery Disease. J Atheroscler Thromb. 2024;31:355–67. 10.5551/jat.64183. 37793811 10.5551/jat.64183 PMC10999719

[44] Simonet WS, Lacey DL, Dunstan CR, Kelley M, Chang MS, Lüthy R, et al. Osteoprotegerin: a novel secreted protein involved in the regulation of bone density. Cell. 1997;89:309–19. 10.1016/S0092-8674(00)80209-3. 9108485 10.1016/s0092-8674(00)80209-3

[45] Samadi S, Sadeghi M, Dashtbayaz RJ, Nezamdoost S, Mohammadpour AH, Jomehzadeh V. Prognostic role of osteoprotegerin and risk of coronary artery calcification: a systematic review and meta-analysis. Biomark Med. 2023;17:171–80. 10.2217/bmm-2022-0621. 37097006 10.2217/bmm-2022-0621

[46] Jono S, Ikari Y, Shioi A, Mori K, Miki T, Hara K, Nishizawa Y. Serum osteoprotegerin levels are associated with the presence and severity of coronary artery disease. Circulation. 2002;106:1192–4. 10.1161/01.cir.0000031524.49139.29. 12208791 10.1161/01.cir.0000031524.49139.29

[47] Jono S, Otsuki S, Higashikuni Y, Shioi A, Mori K, Hara K, et al. Serum osteoprotegerin levels and long-term prognosis in subjects with stable coronary artery disease. J Thromb Haemost. 2010;8:1170–5. 10.1111/j.1538-7836.2010.03833.x. 20230427 10.1111/j.1538-7836.2010.03833.x

[48] Ma T, Zhao J, Yan Y, Liu J, Zang J, Zhang Y, et al. Plasma osteoprotegerin predicts adverse cardiovascular events in stable coronary artery disease: the PEACE trial. Front Cardiovasc Med. 2023;10:1178153. 10.3389/fcvm.2023.1178153. 37388640 10.3389/fcvm.2023.1178153 PMC10300416

[49] National Library of Medicine - Gene. STAMBP (STAM binding protein). https://www.ncbi.nlm.nih.gov/gene?Db=gene&Cmd=ShowDetailView&TermToSearch=10617

[50] McCullough J, Clague MJ, Urbé S. AMSH is an endosome-associated ubiquitin isopeptidase. J Cell Biol. 2004;166:487–92. 10.1083/jcb.200401141. 15314065 10.1083/jcb.200401141 PMC2172215

[51] Björkenheim A, Sunnefeldt E, Finke K, Smith DR, Fröbert O, Brasier N. Biomarkers of inflammation in sweat after myocardial infarction. Sci Rep. 2025;15:5564. 10.1038/s41598-025-90240-8. 39955425 10.1038/s41598-025-90240-8 PMC11829942

[52] Schmitz T, Harmel E, Raake P, Freuer D, Kirchberger I, Heier M, et al. Association Between Acute Myocardial Infarction Symptoms and Short- and Long-term Mortality After the Event. Can J Cardiol. 2024;40:1355–66. 10.1016/j.cjca.2024.01.019. 38278322 10.1016/j.cjca.2024.01.019

[53] Bertero E, Dudek J, Cochain C, Delgobo M, Ramos G, Gerull B, et al. Immuno-metabolic interfaces in cardiac disease and failure. Cardiovascular Res. 2022;118:37–52. 10.1093/cvr/cvab036. 10.1093/cvr/cvab036 33537710

[54] Zang G-Y, Yin Q, Shao C, Sun Z, Zhang L-L, Xu Y, et al. CD137 signaling aggravates myocardial ischemia-reperfusion injury by inhibiting mitophagy mediated NLRP3 inflammasome activation. J Geriatr Cardiol. 2023;20:223–37. 10.26599/1671-5411.2023.03.004. 37091265 10.26599/1671-5411.2023.03.004 PMC10114197

[55] Zhu X, Chen Y, Cai Y, Hu J. Adenosine deaminase is a risk factor for mortality after discharge in patients with acute myocardial infarction: Long-term clinical follow-up. Heliyon. 2024;10:e38401. 10.1016/j.heliyon.2024.e38401. 39416837 10.1016/j.heliyon.2024.e38401 PMC11481646

[56] Schumacher D, Alampour-Rajabi S, Ponomariov V, Curaj A, Wu Z, Staudt M, et al. Cardiac FGF23: new insights into the role and function of FGF23 after acute myocardial infarction. Cardiovasc Pathol. 2019;40:47–54. 10.1016/j.carpath.2019.02.001. 30852297 10.1016/j.carpath.2019.02.001

[57] Leifheit-Nestler M, Kirchhoff F, Nespor J, Richter B, Soetje B, Klintschar M, et al. Fibroblast growth factor 23 is induced by an activated renin-angiotensin-aldosterone system in cardiac myocytes and promotes the pro-fibrotic crosstalk between cardiac myocytes and fibroblasts. Nephrol Dial Transpl. 2018;33:1722–34. 10.1093/ndt/gfy006. 10.1093/ndt/gfy006 29425341

[58] Schmitz T, Wein B, Heier M, Peters A, Meisinger C, Linseisen J. Baseline fibroblast growth factor 23 is associated with long-term mortality in ST-elevation myocardial infarction-results from the augsburg myocardial infarction registry. Front Cardiovasc Med. 2023;10:1173281. 10.3389/fcvm.2023.1173281. 37600039 10.3389/fcvm.2023.1173281 PMC10436601

[59] Bergmark BA, Udell JA, Morrow DA, Cannon CP, Steen DL, Jarolim P, et al. Association of Fibroblast Growth Factor 23 With Recurrent Cardiovascular Events in Patients After an Acute Coronary Syndrome: A Secondary Analysis of a Randomized Clinical Trial. JAMA Cardiol. 2018;3:473–80. 10.1001/jamacardio.2018.0653. 29710336 10.1001/jamacardio.2018.0653 PMC6128514

[60] Almahmoud MF, Soliman EZ, Bertoni AG, Kestenbaum B, Katz R, Lima JAC, et al. Fibroblast Growth Factor-23 and Heart Failure With Reduced Versus Preserved Ejection Fraction: MESA. J Am Heart Assoc. 2018;7:e008334. 10.1161/JAHA.117.008334. 30371180 10.1161/JAHA.117.008334 PMC6222949

[61] Vergaro G, Del Franco A, Aimo A, Gentile F, Castiglione V, Saponaro F, et al. Intact fibroblast growth factor 23 in heart failure with reduced and mildly reduced ejection fraction. BMC Cardiovasc Disord. 2023;23:433. 10.1186/s12872-023-03441-2. 37658340 10.1186/s12872-023-03441-2 PMC10474676

