# Supplementary material for: Evaluation of the relationship between inflammation and typical chest pain in ST-elevation myocardial infarction
Source: Clin Proteomics. 2026 Mar 11;23:18. doi: 10.1186/s12014-026-09596-2 (PMC13063605; doi:10.1186/s12014-026-09596-2)
Supplement: Supplementary file 1 — Supplementary Material 1 [file 12014_2026_9596_MOESM1_ESM.docx]

**Supplementary data**

**Title: Evaluation of the relationship between inflammation and typical chest pain in ST-elevation myocardial infarction**

Sophia Wolfermann^1^, Timo Schmitz^1^, Philip Raake^2^, Jakob Linseisen^1^, Christa Meisinger^1^

^1^ Epidemiology, Medical Faculty, University of Augsburg, 86156 Augsburg, Germany

^2^University Hospital Augsburg, Department of Cardiology, Respiratory Medicine and Intensive Care, 86156 Augsburg, Germany

**Table S1:** Results of the multivariable logistic regression models analyzing the association between inflammatory plasma proteins (Exposure) and typical chest pain symptoms at acute myocardial infarction. All models were adjusted for sex, age, renal function, diabetes and acute infection.

| **Parameter (short)** | **Parameter (long)** | **Odds ratio** | **95% CI** | **P value** | **FDR-adjusted P value** | **Number of measures below limit of detection (%)** |
| --- | --- | --- | --- | --- | --- | --- |
| 4E-BP1 | Eukaryotic translation initiation factor 4E-binding protein 1 (4E-BP1) | 0.61 | 0.42-0.88 | 0.008 | 0.055 | 0.732 |
| ADA | Adenosine Deaminase (ADA) | 0.51 | 0.32-0.79 | 0.003 | 0.028 | 0.732 |
| ARTN | Artemin (ARTN) | 0.86 | 0.61-1.20 | 0.381 | 0.665 | 18.78 |
| AXIN1 | Axin-1 (AXIN1) | 0.92 | 0.63-1.36 | 0.679 | 0.873 | 2.683 |
| Beta-NGF | Beta-nerve growth factor (Beta-NGF) | - | - | - | - | 98.78 |
| CASP-8 | Caspase-8 (CASP-8 ) | 0.61 | 0.40-0.95 | 0.025 | 0.109 | 1.22 |
| CCL11 | Eotaxin (CCL11) | 0.74 | 0.51-1.08 | 0.120 | 0.309 | 0.732 |
| CCL19 | C-C motif chemokine 19 (CCL19) | 0.92 | 0.64-1.33 | 0.642 | 0.865 | 0.732 |
| CCL20 | C-C motif chemokine 20 (CCL20) | 0.81 | 0.57-1.17 | 0.253 | 0.542 | 0.732 |
| CCL23 | C-C motif chemokine 23 (CCL23) | 0.88 | 0.60-1.30 | 0.511 | 0.798 | 0.732 |
| CCL25 | C-C motif chemokine 25 (CCL25) | 0.71 | 0.47-1.07 | 0.105 | 0.292 | 0.732 |
| CCL28 | C-C motif chemokine 28 (CCL28) | 1.07 | 0.76-1.50 | 0.684 | 0.873 | 2.439 |
| CCL3 | C-C motif chemokine 3 (CCL3) | 0.73 | 0.50-1.05 | 0.088 | 0.267 | 0.732 |
| CCL4 | C-C motif chemokine 4 (CCL4 ) | 0.87 | 0.62-1.25 | 0.452 | 0.754 | 0.732 |
| CD244 | Natural killer cell receptor 2B4 (CD244) | 1.06 | 0.72-1.56 | 0.781 | 0.928 | 0.732 |
| CD40 | CD40L receptor (CD40) | 0.46 | 0.29-0.71 | <0.001 | 0.012 | 0.732 |
| CD5 | T-cell surface glycoprotein CD5 (CD5) | 0.80 | 0.49-1.29 | 0.360 | 0.648 | 0.732 |
| CD6 | T cell surface glycoprotein CD6 isoform (CD6) | 0.95 | 0.63-1.43 | 0.821 | 0.944 | 0.732 |
| CD8A | T-cell surface glycoprotein CD8 alpha chain (CD8A) | 1.35 | 0.92-2.00 | 0.126 | 0.314 | 0.732 |
| CDCP1 | CUB domain-containing protein 1 (CDCP1) | 0.85 | 0.58-1.24 | 0.395 | 0.673 | 0.732 |
| CSF-1 | Macrophage colony-stimulating factor 1 (CSF-1) | 0.87 | 0.54-1.39 | 0.553 | 0.798 | 0.732 |
| CST5 | Cystatin D (CST5) | 0.42 | 0.26-0.67 | <0.001 | 0.012 | 0.732 |
| CX3CL1 | Fractalkine (CX3CL1 ) | 0.60 | 0.39-0.89 | 0.014 | 0.076 | 0.732 |
| CXCL1 | C-X-C motif chemokine 1 (CXCL1) | 1.00 | 0.67-1.48 | 0.990 | 0.990 | 0.732 |
| CXCL10 | C-X-C motif chemokine 10 (CXCL10 ) | 0.96 | 0.63-1.49 | 0.870 | 0.946 | 0.976 |
| CXCL11 | C-X-C motif chemokine 11 (CXCL11) | 0.82 | 0.57-1.19 | 0.282 | 0.569 | 0.732 |
| CXCL5 | C-X-C motif chemokine 5 (CXCL5 ) | 1.18 | 0.84-1.64 | 0.336 | 0.631 | 0.732 |
| CXCL6 | C-X-C motif chemokine 6 (CXCL6) | 1.00 | 0.69-1.46 | 0.987 | 0.990 | 0.732 |
| CXCL9 | C-X-C motif chemokine 9 (CXCL9 ) | 0.97 | 0.66-1.43 | 0.862 | 0.946 | 0.732 |
| DNER | Delta and Notch-like epidermal growth factor-related receptor (DNER) | 0.88 | 0.60-1.29 | 0.510 | 0.798 | 0.732 |
| EN-RAGE | Protein S100-A12 (EN-RAGE ) | 0.92 | 0.65-1.29 | 0.612 | 0.850 | 0.732 |
| FGF-19 | Fibroblast growth factor 19 (FGF-19) | 0.75 | 0.52-1.07 | 0.113 | 0.302 | 0.732 |
| FGF-21 | Fibroblast growth factor 21 (FGF-21) | 0.50 | 0.34-0.72 | <0.001 | 0.012 | 0.732 |
| FGF-23 | Fibroblast growth factor 23 (FGF-23) | - | - | - | - | 62.683 |
| FGF-5 | Fibroblast growth factor 5 (FGF-5) | 0.94 | 0.66-1.35 | 0.718 | 0.897 | 5.122 |
| Flt3L | Fms-related tyrosine kinase 3 ligand (Flt3L) | 1.08 | 0.75-1.54 | 0.687 | 0.873 | 0.732 |
| GDNF | Glial cell line-derived neurotrophic factor (GDNF) | 0.74 | 0.49-1.11 | 0.139 | 0.336 | 12.439 |
| HGF | Hepatocyte growth factor (HGF) | 1.08 | 0.77-1.48 | 0.646 | 0.865 | 0.732 |
| IFN-gamma | Interferon gamma (IFN-gamma) | 1.31 | 0.83-2.11 | 0.250 | 0.542 | 1.22 |
| IL-1 alpha | Interleukin-1 alpha (IL-1 alpha) | - | - | - | - | 94.634 |
| IL-10RA | Interleukin-10 receptor subunit alpha (IL-10RA) | - | - | - | - | 37.561 |
| IL-10RB | Interleukin-10 receptor subunit beta (IL-10RB) | 0.94 | 0.63-1.40 | 0.756 | 0.915 | 0.732 |
| IL-12B | Interleukin-12 subunit beta (IL-12B) | 0.70 | 0.46-1.05 | 0.092 | 0.267 | 0.732 |
| IL-15RA | Interleukin-15 receptor subunit alpha (IL-15RA) | 0.58 | 0.37-0.90 | 0.014 | 0.076 | 0.976 |
| IL-17A | Interleukin-17A (IL-17A) | 0.75 | 0.48-1.18 | 0.198 | 0.463 | 23.902 |
| IL-17C | Interleukin-17C (IL-17C) | 0.71 | 0.48-1.06 | 0.089 | 0.267 | 0.732 |
| IL-18R1 | Interleukin-18 receptor 1 (IL-18R1) | 0.66 | 0.45-0.96 | 0.030 | 0.117 | 0.732 |
| IL-20 | Interleukin-20 (IL-20) | - | - | - | - | 92.927 |
| IL-20RA | Interleukin-20 receptor subunit alpha (IL-20RA) | - | - | - | - | 61.463 |
| IL-22 RA1 | Interleukin-22 receptor subunit alpha-1 (IL-22 RA1) | - | - | - | - | 93.171 |
| IL-24 | Interleukin-24 (IL-24) | - | - | - | - | 75.854 |
| IL-2RB | Interleukin-2 receptor subunit beta (IL-2RB) | - | - | - | - | 91.951 |
| IL10 | Interleukin-10 (IL10) | 0.83 | 0.58-1.21 | 0.323 | 0.620 | 0.732 |
| IL13 | Interleukin-13 (IL-13) | - | - | - | - | 91.707 |
| IL18 | Interleukin-18 (IL-18) | 0.89 | 0.61-1.30 | 0.540 | 0.798 | 0.732 |
| IL2 | Interleukin-2 (IL-2) | - | - | - | - | 98.537 |
| IL33 | Interleukin-33 (IL-33) | - | - | - | - | 96.829 |
| IL4 | Interleukin-4 (IL-4) | - | - | - | - | 89.268 |
| IL5 | Interleukin-5 (IL5) | - | - | - | - | 82.439 |
| IL6 | Interleukin-6 (IL6) | 0.57 | 0.40-0.80 | 0.001 | 0.025 | 0.732 |
| IL7 | Interleukin-7 (IL-7) | 0.97 | 0.68-1.40 | 0.889 | 0.952 | 0.732 |
| IL8 | Interleukin-8 (IL-8) | 0.57 | 0.39-0.82 | 0.002 | 0.028 | 0.732 |
| LAP TGF-beta-1 | Latency-associated peptide transforming growth factor beta-1 (LAP TGF-beta-1) | 0.95 | 0.66-1.37 | 0.792 | 0.928 | 0.732 |
| LIF | Leukemia inhibitory factor (LIF) | - | - | - | - | 76.585 |
| LIF-R | Leukemia inhibitory factor receptor (LIF-R) | 0.63 | 0.42-0.93 | 0.021 | 0.105 | 0.732 |
| MCP-1 | Monocyte chemotactic protein 1 (MCP-1) | 0.73 | 0.51-1.04 | 0.076 | 0.253 | 0.732 |
| MCP-2 | Monocyte chemotactic protein 2 (MCP-2) | 1.06 | 0.73-1.53 | 0.753 | 0.915 | 0.732 |
| MCP-3 | Monocyte chemotactic protein 3 (MCP-3) | 0.96 | 0.66-1.42 | 0.849 | 0.946 | 17.805 |
| MCP-4 | Monocyte chemotactic protein 4 (MCP-4) | 1.22 | 0.85-1.77 | 0.288 | 0.569 | 0.732 |
| MMP-1 | Matrix metalloproteinase-1 (MMP-1) | 1.04 | 0.72-1.47 | 0.830 | 0.944 | 0.976 |
| MMP-10 | Matrix metalloproteinase-10 (MMP-10) | 0.70 | 0.49-1.01 | 0.052 | 0.196 | 0.732 |
| NRTN | Neurturin (NRTN) | - | - | - | - | 51.707 |
| NT-3 | Neurotrophin-3 (NT-3) | 0.82 | 0.54-1.26 | 0.363 | 0.648 | 18.537 |
| OPG | Osteoprotegerin (OPG) | 0.58 | 0.40-0.84 | 0.005 | 0.034 | 0.732 |
| OSM | Oncostatin-M (OSM) | 1.12 | 0.78-1.61 | 0.539 | 0.798 | 0.732 |
| PD-L1 | Programmed cell death 1 ligand 1 (PD-L1) | 0.56 | 0.38-0.83 | 0.003 | 0.029 | 0.732 |
| SCF | Stem cell factor (SCF) | 1.12 | 0.74-1.66 | 0.586 | 0.829 | 0.732 |
| SIRT2 | SIR2-like protein 2 (SIRT2) | 0.58 | 0.38-0.88 | 0.010 | 0.060 | 12.195 |
| SLAMF1 | Signaling lymphocytic activation molecule (SLAMF1) | 0.69 | 0.46-1.04 | 0.078 | 0.253 | 1.22 |
| ST1A1 | Sulfotransferase 1A1 (ST1A1) | 0.70 | 0.49-1.02 | 0.056 | 0.199 | 5.61 |
| STAMBP | STAM-binding protein (STAMPB) | 0.50 | 0.32-0.78 | 0.002 | 0.028 | 0.732 |
| TGF-alpha | Transforming growth factor alpha (TGF-alpha) | 1.26 | 0.88-1.85 | 0.213 | 0.483 | 0.732 |
| TNF | Tumor necrosis factor (TNF) | 0.61 | 0.39-0.95 | 0.029 | 0.117 | 0.732 |
| TNFB | TNF-beta (TNFB) | 1.01 | 0.68-1.50 | 0.953 | 0.983 | 0.732 |
| TNFRSF9 | Tumor necrosis factor receptor superfamily member 9 (TNFRSF9) | 0.52 | 0.33-0.80 | 0.003 | 0.029 | 0.732 |
| TNFSF14 | Tumor necrosis factor ligand superfamily member 14 (TNFSF14 ) | 1.60 | 1.08-2.43 | 0.022 | 0.105 | 0.732 |
| TRAIL | TNF-related apoptosis-inducing ligand (TRAIL) | 1.01 | 0.67-1.50 | 0.957 | 0.983 | 0.732 |
| TRANCE | TNF-related activation-induced cytokine (TRANCE) | 1.22 | 0.84-1.77 | 0.286 | 0.569 | 0.732 |
| TSLP | Thymic stromal lymphopoietin (TSLP) | - | - | - | - | 93.171 |
| TWEAK | Tumor necrosis factor (Ligand) superfamily, member 12 (TWEAK) | 1.12 | 0.81-1.56 | 0.486 | 0.793 | 0.732 |
| uPA | Urokinase-type plasminogen activator (uPA) | 0.89 | 0.60-1.30 | 0.548 | 0.798 | 0.732 |
| VEGFA | Vascular endothelial growth factor A (VEGF-A) | 0.98 | 0.67-1.48 | 0.934 | 0.983 | 0.732 |

**Table S2:** Median (IQR) values stratified for chest pain group and compared with U-test. Parameters with more than 25% observation below LoD were included.

| **Parameter** | **Typcial chest pain (Median (IQR)** | **No typcial chest pain (Median (IQR)** | **P Value**  **(U-Test)** | **Number of observations < LoD (%)** |
| --- | --- | --- | --- | --- |
| 4E-BP1 | 8.69 (8.20-8.88) | 9.48 (8.55-9.51) | <0.001 | 0.7 |
| ADA | 6.46 (6.23-6.58) | 6.76 (6.45-6.95) | <0.001 | 0.7 |
| ARTN | 2.62 (2.18-2.61) | 2.72 (2.07-2.67) | 0.429 | 18.8 |
| AXIN1 | 3.74 (3.38-3.82) | 3.96 (3.59-3.92) | 0.162 | 2.7 |
| Beta-NGF | 0.23 (0.18-0.24) | 0.26 (0.19-0.28) | 0.081 | 98.8 |
| CASP-8 | 3.25 (2.95-3.44) | 3.57 (3.27-3.78) | <0.001 | 1.2 |
| CCL11 | 8.78 (8.36-8.77) | 9.00 (8.56-8.92) | 0.070 | 0.7 |
| CCL19 | 9.11 (8.57-9.26) | 9.25 (8.68-9.53) | 0.228 | 0.7 |
| CCL20 | 7.46 (6.86-7.66) | 7.86 (7.04-8.21) | 0.028 | 0.7 |
| CCL23 | 11.14 (10.71-11.21) | 11.40 (11.11-11.59) | 0.003 | 0.7 |
| CCL25 | 7.33 (6.87-7.32) | 7.51 (6.98-7.67) | 0.013 | 0.7 |
| CCL28 | 5.91 (4.58-5.88) | 6.30 (4.27-5.89) | 0.773 | 2.4 |
| CCL3 | 7.30 (6.82-7.34) | 7.66 (7.21-7.71) | 0.003 | 0.7 |
| CCL4 | 7.60 (7.10-7.70) | 7.87 (7.14-7.84) | 0.340 | 0.7 |
| CD244 | 6.59 (6.36-6.64) | 6.65 (6.48-6.67) | 0.496 | 0.7 |
| CD40 | 11.92 (11.64-11.96) | 12.30 (12.00-12.45) | <0.001 | 0.7 |
| CD5 | 6.69 (6.41-6.71) | 6.86 (6.56-6.90) | 0.010 | 0.7 |
| CD6 | 6.36 (5.99-6.36) | 6.24 (6.03-6.26) | 0.175 | 0.7 |
| CD8A | 10.23 (9.83-10.28) | 10.35 (9.46-10.16) | 0.761 | 0.7 |
| CDCP1 | 3.96 (3.53-3.96) | 4.12 (3.76-4.27) | 0.011 | 0.7 |
| CSF-1 | 10.83 (10.69-10.83) | 10.98 (10.78-10.91) | 0.003 | 0.7 |
| CST5 | 6.35 (5.95-6.36) | 6.92 (6.42-7.04) | <0.001 | 0.7 |
| CX3CL1 | 4.88 (4.51-4.89) | 5.16 (4.93-5.30) | <0.001 | 0.7 |
| CXCL1 | 10.23 (9.87-10.27) | 10.38 (9.91-10.38) | 0.207 | 0.7 |
| CXCL10 | 9.54 (9.01-9.67) | 9.86 (9.08-9.88) | 0.135 | 1.0 |
| CXCL11 | 8.02 (7.38-8.12) | 8.12 (7.80-8.39) | 0.100 | 0.7 |
| CXCL5 | 11.95 (11.19-11.91) | 11.66 (11.09-11.72) | 0.272 | 0.7 |
| CXCL6 | 9.67 (9.20-9.68) | 9.79 (9.40-9.87) | 0.142 | 0.7 |
| CXCL9 | 8.99 (8.14-9.08) | 9.61 (8.47-9.40) | 0.095 | 0.7 |
| DNER | 9.27 (9.04-9.26) | 9.25 (9.00-9.17) | 0.345 | 0.7 |
| EN-RAGE | 6.24 (5.58-6.30) | 6.54 (6.03-6.58) | 0.055 | 0.7 |
| FGF-19 | 9.10 (8.42-9.09) | 9.39 (8.81-9.48) | 0.016 | 0.7 |
| FGF-21 | 7.39 (6.41-7.47) | 8.71 (7.24-8.73) | <0.001 | 0.7 |
| FGF-23 | 1.30 (0.98-1.53) | 2.34 (1.32-2.65) | <0.001 | 62.7 |
| FGF-5 | 3.24 (2.86-3.43) | 3.22 (2.94-3.43) | 0.679 | 5.1 |
| Flt3L | 9.73 (9.39-9.74) | 9.81 (9.37-9.79) | 0.696 | 0.7 |
| GDNF | 3.00 (2.66-3.04) | 3.15 (2.82-3.18) | 0.018 | 12.4 |
| HGF | 13.50 (12.68-13.14) | 13.66 (12.33-13.12) | 0.544 | 0.7 |
| IFN-gamma | 6.62 (5.95-6.82) | 6.60 (5.73-6.55) | 0.440 | 1.2 |
| IL-1 alpha | -1.14 (-1.27--1.05) | -1.07 (-1.21--1.07) | 0.038 | 94.6 |
| IL10 | 5.68 (4.92-5.97) | 6.18 (4.98-6.64) | 0.075 | 0.7 |
| IL-10RA | 2.05 (1.90-2.28) | 2.06 (1.92-2.16) | 0.998 | 37.6 |
| IL-10RB | 7.82 (7.60-7.82) | 7.94 (7.73-8.02) | 0.006 | 0.7 |
| IL-12B | 7.55 (7.08-7.54) | 7.95 (7.44-7.77) | 0.005 | 0.7 |
| IL13 | 1.18 (0.99-1.31) | 1.29 (0.98-1.28) | 0.394 | 91.7 |
| IL-15RA | 2.72 (2.54-2.78) | 2.92 (2.71-3.01) | <0.001 | 1 |
| IL-17A | 3.50 (3.18-3.62) | 3.67 (3.48-3.82) | 0.010 | 23.9 |
| IL-17C | 3.89 (3.41-4.08) | 4.29 (3.80-4.59) | <0.001 | 0.7 |
| IL18 | 9.87 (9.61-9.94) | 10.10 (9.67-10.16) | 0.064 | 0.7 |
| IL-18R1 | 8.96 (8.68-8.99) | 9.20 (8.78-9.18) | 0.024 | 0.7 |
| IL2 | 1.86 (1.73-1.91) | 1.92 (1.77-1.86) | 0.217 | 98.5 |
| IL-20 | 1.78 (1.68-1.83) | 1.91 (1.79-1.90) | <0.001 | 92.9 |
| IL-20RA | 2.12 (1.95-2.20) | 2.13 (2.00-2.18) | 0.412 | 61.5 |
| IL-22 RA1 | 2.47 (2.20-2.50) | 2.54 (2.28-2.61) | 0.167 | 93.2 |
| IL-24 | 2.70 (2.40-2.78) | 2.78 (2.60-2.94) | 0.057 | 75.9 |
| IL-2RB | 2.03 (1.88-2.12) | 2.09 (1.93-2.15) | 0.225 | 92.0 |
| IL33 | 2.07 (1.91-2.11) | 2.17 (2.02-2.20) | 0.003 | 96.8 |
| IL4 | 1.90 (1.64-2.01) | 1.84 (1.49-1.93) | 0.410 | 89.3 |
| IL5 | 1.71 (1.52-1.95) | 1.75 (1.52-1.90) | 0.433 | 82.4 |
| IL6 | 5.56 (4.95-5.93) | 6.68 (5.86-7.19) | <0.001 | 0.7 |
| IL7 | 4.37 (3.76-4.36) | 4.37 (3.96-4.35) | 0.937 | 0.7 |
| IL8 | 6.08 (5.25-6.15) | 6.79 (6.11-6.98) | <0.001 | 0.7 |
| LAP TGF-beta-1 | 8.27 (7.93-8.30) | 8.38 (8.09-8.40) | 0.183 | 0.7 |
| LIF | 0.78 (0.58-0.94) | 0.98 (0.77-1.13) | 0.002 | 76.6 |
| LIF-R | 4.41 (4.22-4.45) | 4.67 (4.49-4.69) | <0.001 | 0.7 |
| MCP-1 | 12.69 (12.18-12.77) | 13.10 (12.45-13.06) | 0.049 | 0.7 |
| MCP-2 | 10.29 (9.74-10.27) | 10.33 (9.71-10.27) | 0.975 | 0.7 |
| MCP-3 | 2.62 (2.23-2.75) | 2.82 (2.41-3.04) | 0.027 | 17.8 |
| MCP-4 | 13.91 (13.33-13.96) | 13.93 (13.18-13.83) | 0.426 | 0.7 |
| MMP-1 | 15.44 (14.62-15.28) | 15.56 (14.41-15.34) | 0.640 | 1.0 |
| MMP-10 | 9.77 (9.35-9.81) | 10.10 (9.62-10.18) | 0.003 | 0.7 |
| NRTN | 2.09 (1.85-2.16) | 2.07 (1.85-2.16) | 0.787 | 51.7 |
| NT-3 | 3.00 (2.77-3.03) | 3.00 (2.69-3.08) | 0.577 | 18.5 |
| OPG | 11.13 (10.83-11.20) | 11.74 (11.24-11.65) | <0.001 | 0.7 |
| OSM | 6.60 (5.92-6.69) | 6.75 (6.14-6.82) | 0.418 | 0.7 |
| PD-L1 | 6.50 (6.26-6.56) | 6.85 (6.57-6.95) | <0.001 | 0.7 |
| SCF | 9.70 (9.29-9.59) | 9.70 (9.35-9.63) | 0.642 | 0.7 |
| SIRT2 | 4.10 (3.63-4.41) | 4.75 (4.01-4.84) | 0.002 | 12.2 |
| SLAMF1 | 3.60 (3.32-3.66) | 3.90 (3.64-4.01) | <0.001 | 1.2 |
| ST1A1 | 3.16 (2.56-3.32) | 3.55 (2.94-3.87) | 0.006 | 5.6 |
| STAMBP | 5.01 (4.67-5.25) | 5.39 (5.01-5.63) | <0.001 | 0.7 |
| TGF-alpha | 4.33 (3.79-4.52) | 4.48 (3.98-4.62) | 0.455 | 0.7 |
| TNF | 4.52 (4.22-4.60) | 4.78 (4.61-4.87) | <0.001 | 0.7 |
| TNFB | 5.19 (4.84-5.17) | 5.07 (4.77-5.10) | 0.254 | 0.7 |
| TNFRSF9 | 6.68 (6.34-6.76) | 7.13 (6.71-7.33) | <0.001 | 0.7 |
| TNFSF14 | 6.53 (6.02-6.68) | 6.14 (5.70-6.51) | 0.144 | 0.7 |
| TRAIL | 8.32 (8.05-8.27) | 8.15 (7.91-8.15) | 0.183 | 0.7 |
| TRANCE | 5.28 (4.82-5.19) | 5.00 (4.55-4.92) | 0.020 | 0.7 |
| TSLP | 2.06 (1.80-2.09) | 1.99 (1.78-2.06) | 0.926 | 93.2 |
| TWEAK | 11.11 (10.28-11.02) | 11.09 (10.07-10.84) | 0.435 | 0.7 |
| uPA | 10.32 (10.08-10.32) | 10.35 (10.13-10.41) | 0.250 | 0.7 |
| VEGFA | 11.57 (11.23-11.74) | 11.84 (11.46-12.02) | 0.022 | 0.7 |


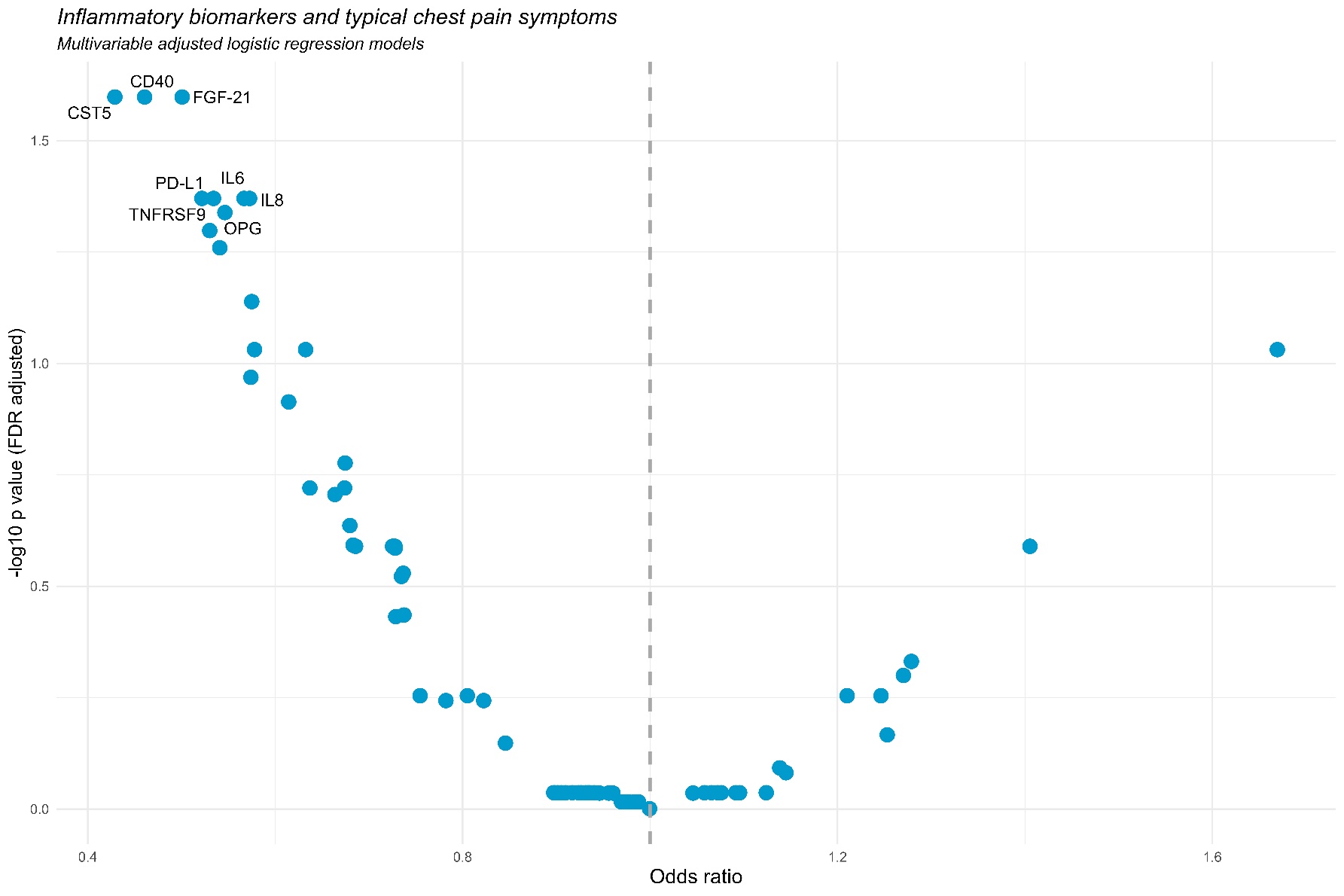


***Figure S1:*** *Results of the sensitivity analysis. The logistic regression models were adjusted for sex, age, renal function, diabetes, acute infection (CRP-values > 10 mg/dL) and additionally for troponin I quantiles. P-values were FDR-adjusted. Names of the markers are presented for all markers with FDR-adjusted p-values below 0.05*
